# Effects of Replacing Oat Hay with Peanut Hull Depolymerization Product on Growth Performance, Serum Biochemical Parameters, and Rumen Fermentation in Holstein Dairy Bulls

**DOI:** 10.3390/ani16121864

**Published:** 2026-06-17

**Authors:** Zixin Qiao, Yihui Zhang, Yujie Feng, Yucong Wang, Shujing Tian, Meng Song, Yongjiu Huo, Dongyuan Zhang, Kexin Wang, Guoqi Zhao

**Affiliations:** 1 College of Animal Science and Technology, Yangzhou University, Yangzhou 225009, China; 15530516171@163.com (Z.Q.);; 2 Experimental Farm of Yangzhou University, Yangzhou University, Yangzhou 225009, China; yhz@yzu.edu.cn; 3 Zhongke Kangyuan (Tangshan) Biotechnology Co., Ltd., Gangbin Street Office Building, Tangshan Haigang Development Zone, Tangshan 063611, China; 4 College of Life Science, Longyan University, Longyan 364012, China

**Keywords:** peanut hull depolymerization product, Holstein dairy bulls, growth performance, rumen fermentation, serum biochemistry, antioxidant status

## Abstract

Alternative roughage resources are increasingly important for sustainable ruminant production. This study evaluated the effects of replacing oat hay with a peanut hull depolymerization product (PHDP) on growth performance, apparent nutrient digestibility, serum biochemical variables, antioxidant status, and rumen fermentation characteristics in Holstein dairy bulls. Thirty-six Holstein dairy bulls (18–22 months old) were randomly assigned to four dietary treatments in a completely randomized design (*n* = 9 per treatment): T0, a basal diet containing 24% oat hay; T1, T2, and T3, in which 25%, 50%, and 75% of the oat hay was replaced with PHDP, respectively. The trial comprised a 14 d adaptation period and a 60 d measurement period. Growth performance and rumen fermentation data were analyzed using one-way ANOVA with linear and quadratic contrasts, while serum biochemical and antioxidant variables were analyzed using ANCOVA with baseline values as covariates. PHDP substitution significantly affected average daily gain (ADG), dry matter intake (DMI), and feed conversion ratio (FCR). Compared with the control group, the 75% replacement group showed greater ADG and lower FCR, whereas DMI was lower in T1 than in the other groups. Ruminal pH was increased in the 75% replacement group, whereas ammonia nitrogen concentration, major volatile fatty acid (VFA) concentrations, total VFA concentration, and apparent nutrient digestibility were not significantly affected by treatment. Most hematological and serum biochemical variables were not markedly influenced by PHDP substitution. However, total antioxidant capacity at d 60 was higher in T2 and T3 than in T1, catalase activity at d 30 was higher in T3 than in T0, and serum insulin-like growth factor-1 concentration at d 60 was highest in T3. In conclusion, PHDP could partially replace oat hay in diets for Holstein dairy bulls without impairing rumen fermentation, apparent nutrient digestibility, or major blood biochemical indicators. The 75% replacement level showed the most favorable response by improving growth performance and feed efficiency while supporting antioxidant status and serum IGF-1 concentration.

## Simple Summary

Developing alternative roughage resources is an important approach to improving feed utilization efficiency in dairy bull fattening systems. Peanut shell-based by-products are abundant but have limited direct feeding value due to their high fiber and lignin content. This study evaluated whether a peanut hull depolymerization product derived from peanut shell-based materials could replace oat hay in diets for Holstein dairy bulls. The results showed that this product did not impair rumen fermentation, apparent nutrient digestibility, or major blood biochemical indicators. The 75% replacement level improved growth performance and feed efficiency and was associated with improved antioxidant capacity and higher serum insulin-like growth factor-1 concentration. These findings suggest that this processed fibrous product may be a useful alternative roughage resource for dairy bull fattening diets.

## 1. Introduction

Efficient utilization of alternative forage resources has become a major focus in modern ruminant production. In ruminant diets, adequate roughage is required to maintain normal rumen function and support stable animal performance [1]. Therefore, alternative feeds and agro-industrial by-products have received increasing attention as partial substitutes for conventional forages [2,3,4]. In beef cattle production, roughage source can influence feed intake, nutrient utilization, rumen fermentation, and feeding efficiency. Oat hay is a valuable roughage commonly used in ruminant diets, and increasing its dietary proportion has been reported to improve fiber digestibility and help stabilize the ruminal environment [5].

Peanut shells are widely available agricultural by-products in peanut-producing regions, including China, but their use as feed ingredients is constrained by low nutritive value [6,7]. Their high structural carbohydrate and lignin contents, together with relatively low crude protein concentration, limit digestibility and reduce their direct feeding value [8,9]. In the rumen, lignin and lignin–carbohydrate cross-linking hinder microbial access to plant cell wall polysaccharides, thereby restricting fiber degradation [10,11,12]. Accordingly, physical, chemical, and biological processing methods have been explored to improve the feeding value of lignocellulosic by-products. For peanut hulls, chemical treatment, grinding, and alkali treatment have been shown to improve digestibility and alter ruminal responses [13,14]. Similar improvements in digestibility and rumen fermentation have also been reported for other treated fibrous by-products, such as fungal- or urea-treated oil palm fronds [15,16]. However, the utilization of these materials in animals depends on factors such as their processing methods, inclusion level, and mixing methods in the diet [17,18].

Taken together, previous studies suggest that processing can improve the nutritional value of fibrous by-products, but whether a depolymerized peanut shell-derived product can replace conventional forage in dairy bull fattening diets remains unclear. The peanut hull depolymerization product (PHDP) used in this study was derived from peanut shell-based raw materials, which are abundant but generally limited in direct feeding value [6,8]. Compared with untreated peanut shells or other unprocessed fibrous residues, PHDP may provide greater substrate availability for rumen microorganisms and therefore has potential as a partial substitute for oat hay. At present, limited information is available on the use of peanut shell-derived depolymerized products in dairy bull diets, particularly regarding growth performance, apparent nutrient digestibility, serum biochemical and antioxidant responses, and rumen fermentation characteristics. Therefore, this study evaluated the effects of replacing oat hay with increasing levels of PHDP on growth performance, apparent nutrient digestibility, serum biochemical parameters, antioxidant capacity, and rumen fermentation characteristics in Holstein dairy bulls. We hypothesized that partial replacement of oat hay with PHDP would maintain normal growth performance and rumen fermentation stability, whereas an appropriate substitution level might improve nutrient utilization and metabolic status.

## 2. Materials and Methods

### 2.1. Experimental Design

The experiment was conducted from July to September 2025 at the experimental farm of Yangzhou University, Gaoyou, Jiangsu, China. A total of 36 healthy Holstein dairy bulls aged 18 to 22 months were selected for the study. To maximize genetic homogeneity and minimize individual baseline variations, all experimental bulls were selected from a paternal half-sib family (sharing the same sire), with similar initial body weight and age. The animals were randomly assigned to one of four dietary treatments (*n* = 9 per treatment) in a completely randomized design. The treatments were as follows: T0 (control), a basal diet containing 24% oat hay on a dry matter (DM) basis; T1, T2, and T3, in which 25%, 50%, and 75% of the oat hay was replaced with PHDP, respectively.

The PHDP was manufactured from raw peanut hulls via sequential processes, including crushing, mild dilute acid/alkali pretreatment, microbial fermentation, and extrusion puffing, which are processing treatments, to improve its biological nutritional value. On a dry matter basis, the raw untreated peanut hulls contained 99.2% DM, 5.7% CP, 75.7% NDF, and 63.9% ADF, whereas the processed PHDP utilized in this study contained 92.9% DM, 11.8% CP, 62.9% NDF, and 59.5% ADF.

The treatments were as follows: T0 (control), a basal diet containing 24% oat hay on a dry matter (DM) basis; T1, T2, and T3, in which 25%, 50%, and 75% of the oat hay was replaced with PHDP, respectively. The experiment comprised a 14 d adaptation period and a 60 d measurement period. During the 14 d adaptation period, PHDP was gradually increased from 0% to the target replacement level (up to 75% of oat hay) to facilitate ruminal adaptation. No feed refusal, sorting behavior, or diarrhea was observed in any treatment group.

### 2.2. Experimental Diets and Management

The experimental diets were formulated to meet or exceed the nutrient requirements of dairy bulls according to NRC (2001) [19]. The diets mainly consisted of corn silage, oat hay, PHDP, and a concentrate supplement. The ingredient composition and nutrient levels of the experimental diets are presented in Table 1.

### 2.3. Sample Collection and Measurements

#### 2.3.1. Feed Sampling and Chemical Analysis

Feed and ort samples were collected regularly at 2-week intervals throughout the 60 d measurement period. Samples collected across the entire experimental period were pooled to form a representative composite sample, dried at 65 °C to constant weight, ground to pass through a 60-mesh sieve, and analyzed for chemical composition.

DM was determined according to GB/T 6435-2014 [20], crude protein (CP) according to GB/T 6432-1994 [21], ether extract (EE) according to GB/T 6433-2006 [22], ash according to GB/T 6438-2007 [23], calcium (Ca) according to GB/T 6436-2002 [24], and phosphorus (P) according to GB/T 6437-2002 [25]. Neutral detergent fiber (NDF) and acid detergent fiber (ADF) were determined using an ANKOM-2000i fiber analyzer (ANKOM Technology, Macedon, NY, USA) according to the method of Van Soest et al. [26].

#### 2.3.2. Growth Performance Measurement

The amount of feed offered daily and orts remaining on the following day were recorded for DMI. Body weight (BW) was measured before the morning feeding on d 0 and d 50. Average daily gain (ADG) and feed conversion ratio (FCR) were calculated as follows:

ADG = (final BW − initial BW)/experimental days

FCR = DMI/ADG

#### 2.3.3. Preparation of Serum Samples

Blood samples were collected from the coccygeal vein before the morning feeding on d 30 and d 60. Samples were centrifuged at 3000× *g* for 15 min at 4 °C, and the harvested serum was stored at −20 °C until analysis.

Routine hematological variables were determined using an automatic veterinary analyzer (VH50, Genrui Biotech Inc., Shenzhen, China). Serum ALT, AST, and antioxidant markers (SOD, MDA, GSH, GSH-PX, T-AOC, and CAT) were quantified via an automatic chemistry analyzer (BS-240VET, Mindray Bio-Medical Electronics Co., Ltd., Shenzhen, China). Serum metabolic profiles (TP, ALB, GLB, GLU, TG, TC, and CRE) were determined colorimetrically using commercial kits (Yangzhou Hengyi Biotechnology Co., Ltd., Yangzhou, China), while serum growth hormone (GH) and insulin-like growth factor-1 (IGF-1) concentrations were quantified via commercial bovine-specific enzyme-linked immunosorbent assay (ELISA) kits from the same supplier, with all reactions measured on a microplate reader (Multiskan GO, Thermo Fisher Scientific Inc., Waltham, MA, USA). All utilized commercial assay kits were pre-validated specifically for bovine serum cross-reactivity and sensitivity according to standard quality control guidelines.

#### 2.3.4. Collection and Analysis of Rumen Fluid and Fecal Samples

On d 60, rumen fluid samples were collected 2 h after the morning feeding using an oral stomach tube. The first 50 mL of rumen fluid was discarded to minimize contamination with saliva. Subsequently, approximately 50 mL of rumen fluid was filtered through four layers of cheesecloth. The pH was measured immediately using a portable pH meter. The remaining sample was mixed with 25% metaphosphoric acid and stored at −20 °C for the determination of volatile fatty acids (VFA) and ammonia nitrogen (NH_3_-N).

VFA concentrations were determined by gas chromatography (GC-9800), and NH_3_-N concentration was measured by colorimetry.

To avoid animal stress associated with total fecal collection, acid-insoluble ash (AIA) was employed as an internal, natural indigestible marker to estimate apparent total-tract nutrient digestibility. Fecal samples (approximately 200 g per animal per day) were collected via rectal grab sampling before the morning feeding during the final 3 consecutive days of both the mid-trial period (d 28–30) and the end-trial period (d 58–60). Fecal samples were thoroughly pooled by individual animal within each specific sampling period and then split into two separate subsamples. The first subsample was treated with 10% H_2_SO_4_ to fix nitrogen and preserve crude protein. The second, non-acidified subsample was directly dried at 65 °C to constant weight and ground through a 60-mesh sieve to determine DM, NDF, ADF, EE, ash, and acid-insoluble ash (AIA) to avoid potential carbohydrate hydrolysis. The concentrations of AIA in both the experimental diets and the non-acidified fecal samples were determined following the classic method of Van Keulen and Young (1977) [27] to calculate the apparent total-tract digestibility of nutrients.

Apparent nutrient digestibility was calculated as follows:

Apparent nutrient digestibility (%) = 100 − 100 × (AIA in diet/AIA in feces) × (nutrient concentration in feces/nutrient concentration in diet).

### 2.4. Statistical Analysis

Data were analyzed using SPSS 27.0 (IBM Corp., Armonk, NY, USA), with each bull considered the experimental unit. Normality and homogeneity of variance were checked prior to analysis. Growth performance, rumen fermentation, apparent nutrient digestibility, and blood routine parameters were analyzed by one-way ANOVA, with linear and quadratic orthogonal contrasts performed to evaluate dose-dependent trends across PHDP substitution levels (0%, 25%, 50%, and 75%). For serum biochemical, antioxidant, and hormonal variables (GH and IGF-1) measured at multiple time points, data from each sampling day were analyzed separately by analysis of covariance (ANCOVA), with the corresponding baseline (d 0) value included as a covariate to adjust for pre-existing differences among individuals, as the primary objective was to evaluate treatment effects at specific physiological milestones (mid-trial d 30 and end-trial d 60) rather than to model temporal trajectories. Treatment means were compared using Tukey’s honest significant difference (HSD) test and are presented as adjusted means with pooled standard error of the mean (SEM). Significance was declared at *p* < 0.05, and a tendency was considered at 0.05 ≤ *p* < 0.10.

Data were analyzed using the following model:

$$Y_{ij}=\mu +T_{i}+\varepsilon_{ij}$$

where *Y_ij_* is the dependent variable, μ is the overall mean, *T_i_* is the fixed effect of the *i*-th dietary treatment (*i* = T0, T1, T2, T3), and ε*_ij_* is the random error term. For serum biochemical, antioxidant, and hormonal parameters, baseline (d 0) values were included as covariates in an ANCOVA framework:

$$Y_{ij}=\mu +T_{i}+\beta \left(X_{ij}-\bar{X}\right)+\varepsilon_{ij}$$

where β is the regression coefficient for the covariate, *X_ij_* is the baseline (d 0) measurement, and $\bar{X}$ is the overall baseline mean. Linear and quadratic contrasts were applied to evaluate dose–response trends across treatment levels.

## 3. Results

### 3.1. Effects of PHDP on Growth Performance in Holstein Dairy Bulls

The effects of PHDP substitution on the growth performance of Holstein dairy bulls are shown in Table 2. The initial and final BW were similar among treatments (*p* > 0.05). However, increasing PHDP substitution levels linearly and quadratically increased ADG and DMI, while linearly decreasing FCR (*p* < 0.01). Specifically, the T3 group achieved the highest ADG and the lowest FCR (*p* < 0.001), whereas a transient decline in ADG and DMI, coupled with an elevated FCR, was observed in the T1 group.

### 3.2. Effects of PHDP on Rumen Fermentation Parameters in Holstein Dairy Bulls

Rumen fermentation parameters are summarized in Table 3. Increasing PHDP substitution levels significantly affected rumen pH and the Acetate/Propionate (A/P) ratio (*p* < 0.05). Specifically, pH and the A/P ratio exhibited significant linear increases (*p* < 0.01), with a notable quadratic response also detected for pH (*p* = 0.017); the highest values for both parameters were observed in the T3 group. In contrast, no significant treatment or trend effects were detected for NH_3_-N, TVFA, or any individual volatile fatty acids (*p* > 0.05).

### 3.3. Effects of PHDP on Hematological Parameters in Holstein Dairy

As shown in Table 4, WBC and Neu counts tended to increase with increasing PHDP substitution levels (0.05 ≤ *p* < 0.10). Trend analysis further revealed significant linear increases for WBC, Lym, and PLT counts (*p* < 0.05), with WBC achieving a precise linear significance (*p* = 0.034). In contrast, no significant overall effects or trend responses were observed for the remaining hematological parameters (*p* > 0.05).

### 3.4. Effects of PHDP on Serum Biochemical Parameters in Holstein Dairy Bulls

#### 3.4.1. Serum Biochemical Parameters

Serum biochemical parameters in Holstein bulls are presented in Table 5. At d 30, no significant treatment effect was observed for TP, ALB, GLB, A/G, GLU, TCHO, TG, or CRE (*p* > 0.05). ALT was lower in T1 than in T0, T2, and T3 (*p* < 0.05), and AST was lower in T1 than in T0 and T3 (*p* < 0.05). At d 60, ALB was higher in T2 than in T0 (*p* < 0.05), whereas no significant treatment effect was observed for the other parameters (*p* > 0.05).

#### 3.4.2. Antioxidant Status Indicators

As presented in Table 6, at d 30, CAT was higher in T1, T2, and T3 than in T0 (*p* < 0.05). At d 60, T-AOC was higher in T2 and T3 than in T1 (*p* < 0.05). No significant treatment effect was observed for the remaining antioxidant parameters (*p* > 0.05).

#### 3.4.3. Hormone Concentrations

As shown in Table 7, GH differed among treatments only at d 30 (*p* < 0.05), with a lower value in T1 than in T3. At d 60, IGF-1 was higher in T3 than in the other groups (*p* < 0.05), whereas no treatment effect was observed at d 30 (*p* > 0.05).

### 3.5. Effects of PHDP on Apparent Nutrient Digestibility in Holstein Dairy Bulls

The apparent digestibility of nutrients is presented in Table 8. No significant differences were observed among treatment groups in the apparent digestibility of DM, NDF, ADF, CP, or EE at either d 30 or d 60 (*p* > 0.05).

## 4. Discussion

Replacing oat hay with PHDP significantly affected growth performance in Holstein dairy bulls. Bulls in T3 (75% replacement) exhibited greater ADG and lower FCR than those in T0, whereas T1 (25% replacement) unexpectedly showed lower ADG, lower DMI, and higher FCR, indicating a pronounced non-linear dose response. Processing methods that disrupt the lignocellulosic matrix can increase substrate accessibility and improve the feeding value of fibrous by-products [10,11,12,13,14,15,16,28]; accordingly, the improved ADG and FCR in T3, together with the absence of reduced DMI relative to the control, suggest that PHDP at high substitution levels enhanced the functional feeding value of the diet. Dietary CP increased numerically from 9.14% in T0 to 10.27% in T3, and ADF similarly varied among treatments (Table 1), reflecting the inherent compositional differences between oat hay and PHDP. The favorable growth response in T3 therefore likely reflects the combined contribution of higher dietary CP supply and the modified fiber profile of this specific total mixed ration, rather than an isolated effect of PHDP substitution alone. Conversely, the depressed ADG and DMI in T1 suggest that low-level PHDP substitution may have disrupted the physical structure of the diet—possibly by reducing physically effective fiber without providing sufficient compensatory fermentable substrate—leading to altered rumen fill and passage dynamics, although this requires further investigation. Overall, the performance response observed across treatments represents a comprehensive diet-level property under the conditions of this study, which is consistent with previous findings that appropriately processed by-product feeds can support ruminant performance when included at adequate levels [2,18,29,30,31].

Rumen fermentation data supported the growth performance results. Apart from ruminal pH, all major and branched-chain VFA concentrations, NH_3_-N, and TVFA concentration were not significantly affected by PHDP substitution. The higher ruminal pH observed in T3, coupled with unchanged TVFA, suggests that high-level PHDP substitution maintained a stable rumen environment without suppressing fermentative activity. A successful roughage substitute must preserve rumen stability while providing sufficient fermentable substrate [32]. In high-concentrate diets, adequate physically effective fiber sustains chewing activity, saliva flow, and buffering capacity, preventing subacute ruminal acidosis [33]. The elevated pH in T3 thus suggests that PHDP retained part of the functional roughage characteristics of oat hay. The altered ruminal fermentation parameters might be attributed to the expanded physical surface area of the processed PHDP fiber matrix alongside the synchronized nitrogen availability provided by the higher dietary CP concentration in the T3 group. Specifically, the processing of raw peanut hulls into PHDP modified rigid structural fiber fractions, which could facilitate microbial attachment. The synchronized availability of processed carbohydrate fractions and nitrogen in PHDP may foster a favorable microenvironment for microbial protein synthesis and post-ruminal nutrient assimilation without altering total VFA yields.

Apparent nutrient digestibility was also not significantly affected by PHDP substitution. Although total-tract apparent digestibility is a relatively broad indicator that may not capture subtle shifts in rumen passage rate, microbial protein synthesis, or post-ruminal nutrient utilization, this stability indicates that PHDP did not impair digestive kinetics. Because the experimental diets were formulated to be comparable in nutrient supply, PHDP may have altered the physical and fermentative characteristics of the fiber fraction without producing a large detectable difference in total-tract digestibility. These findings are consistent with previous studies showing that bio-processed co-products can be incorporated into ruminant diets without impairing nutrient digestibility when dietary fiber supply is adequate [18,30,34].

Most hematological and serum biochemical variables were not significantly affected by PHDP substitution, indicating that replacing oat hay with PHDP did not cause obvious adverse effects on systemic physiological status. The tendency for increased WBC and Neu counts (*p* < 0.10) may reflect a mild immunomodulatory response, although these changes were not accompanied by consistent alterations in RBC-related variables, hemoglobin concentration, hematocrit, or platelet count, and should therefore be interpreted with caution [35]. Serum creatinine remained stable across treatments, and the changes in ALT and AST were not progressive or dose-dependent, suggesting that PHDP substitution did not impose a detectable metabolic, hepatic, or renal burden under the conditions of this study [36]. The antioxidant and hormonal responses provided additional exploratory, albeit not definitive, support for the growth performance findings. T-AOC at d 60 was higher in T2 and T3 than in T1, and CAT activity at d 30 was higher in T3 than in T0 and T1, whereas MDA concentration was not increased following dietary treatment. This pattern suggests that medium to high PHDP substitution may have supported antioxidant defense without promoting lipid peroxidation. While previous studies have linked antioxidant capacity and oxidative stress markers with variation in feed efficiency [37,38,39,40], these parameters in our study primarily serve as exploratory indicators of systemic physiological safety. Serum IGF-1 concentration at d 60 was higher in T3 than in the other groups, which is consistent with the greater ADG and lower FCR observed in this group. Circulating IGF-1 is closely related to nutritional status, anabolic activity, growth rate, and feed efficiency in cattle [41,42,43,44,45,46], and the elevated IGF-1 concentration in T3 may reflect the comprehensive nutritional and anabolic attributes of this specific total mixed ration. However, several serum variables differed among groups at baseline, and GH responses were not sustained across sampling times; therefore, these endocrine and antioxidant results should be strictly regarded as supportive, exploratory physiological observations rather than definitive mechanistic explanations.

Overall, PHDP appeared to function as a processed fibrous feed resource rather than as an untreated peanut shell residue. The present study did not directly measure the physical effectiveness, particle size distribution, extent of lignin modification, rumen microbiota composition, or microbial protein synthesis associated with PHDP feeding. Further studies are warranted to characterize the structural properties and physically effective fiber value of PHDP and to elucidate how this processed fibrous ingredient influences rumen microbial function, nutrient partitioning, and long-term production responses in dairy bulls.

## 5. Conclusions

In the present study, peanut hull depolymerization product (PHDP) could partially replace oat hay in diets for Holstein dairy bulls without impairing rumen fermentation, apparent nutrient digestibility, or major blood biochemical indicators. Under the conditions of this study, the T3 diet showed favorable performance responses, but further studies are needed to confirm the optimal inclusion level and clarify the nutritional mechanisms.

## Figures and Tables

**Table 1:** Ingredient and nutrient composition of the experimental diets (DM basis, %).

Items	T0	T1	T2	T3
**Ingredients**				
Corn silage	53	53	53	53
Oat hay	24	18	12	6
PHDP	0	6	12	18
Corn	6.9	6.9	6.9	6.9
Germ meal	2.1	2.1	2.1	2.1
Barley	1.0	1.0	1.0	1.0
Soybean meal	3.5	3.5	3.5	3.5
Soybean hulls	2.3	2.3	2.3	2.3
DDGS	2.5	2.5	2.5	2.5
Wheat roots	3.5	3.5	3.5	3.5
Premix ^(1)^	1.2	1.2	1.2	1.2
Total	100	100	100	100
**Nutrient levels ^(2)^**				
NEmf, MJ/kg	5.99	5.98	5.96	5.95
CP	9.14	9.23	9.42	10.27
NDF	49.66	49.35	48.34	49.78
ADF	28.13	28.42	29.09	31.61
EE	2.85	3.56	3.23	3.40
Ash	6.13	6.04	6.04	5.69

Abbreviations: PHDP, peanut hull depolymerization product; DDGS, distiller dried grains with soluble; NEmf, net energy value used for fattening diet formulation; CP, crude protein; NDF, neutral detergent fiber; ADF, acid detergent fiber; EE, ether extract. ^(1)^ The premix provided the following per kilogram of diet: vitamin A, 5500 IU; vitamin D, 1200 IU; vitamin E, 20 IU; Ca, 0.8%; P, 0.4%; and NaCl, 0.5%. ^(2)^ NEmf values were calculated according to NRC (2001) [19], whereas all other nutrient levels were chemically analyzed values.

**Table 2:** Effects of PHDP substitution for oat hay on growth performance in Holstein dairy bulls.

Item	Treatment				SEM	*p*-Value		
	T0	T1	T2	T3		Mean	Linear	Quadratic
IBW, kg	587.0	605.7	591.3	583.2	12.10	0.913	0.814	0.585
FBW, kg	635.0	644.0	651.25	669.6	12.09	0.776	0.319	0.842
ADG, kg/d	0.97 ^b^	0.77 ^b^	1.20 ^b^	1.73 ^a^	0.057	<0.001	<0.001	0.003
DMI, kg/d	9.90 ^a^	9.60 ^b^	9.89 ^a^	9.99 ^a^	0.022	<0.001	<0.001	<0.001
FCR	10.20 ^b^	12.46 ^a^	8.24 ^c^	5.77 ^d^	0.024	<0.001	<0.001	<0.001

Abbreviations: IBW, initial body weight; FBW, final body weight; ADG, average daily gain; DMI, dry matter intake; FCR, feed conversion ratio. ^a–d^, Means within a row with different superscripts differ significantly (*p* < 0.05).

**Table 3:** Effects of PHDP substitution for oat hay on rumen fermentation parameters in Holstein dairy bulls.

Items	Treatment				SEM	*p*-Value		
	T0	T1	T2	T3		Mean	Linear	Quadratic
pH	6.42 ^b^	6.42 ^b^	6.31 ^b^	6.86 ^a^	0.055	0.007	0.006	0.017
NH_3_-N, mg/dL	23.52	23.66	27.62	23.97	2.529	0.651	0.670	0.502
Acetate, mmol/L	53.48	52.27	56.1	57.08	2.254	0.817	0.405	0.750
Propionate, mmol/L	19.19	18.23	18.88	17.82	1.000	0.955	0.692	0.979
A/P	2.79 ^b^	2.88 ^ab^	3.01 ^ab^	3.31 ^a^	0.073	0.038	0.006	0.369
Isobutyrate, mmol/L	0.67	0.56	0.72	0.67	0.029	0.263	0.531	0.554
Butyrate, mmol/L	34.4	34.39	39.11	30.01	1.042	0.145	0.476	0.099
Isovalerate, mmol/L	1.28	1.2	1.21	1.31	0.076	0.947	0.889	0.563
Valerate, mmol/L	1.13	1.27	1.31	1.3	0.089	0.874	0.512	0.685
Hexanoate, mmol/L	0.44	0.62	0.62	0.69	0.045	0.259	0.082	0.575
TVFA, mmol/L	110.58	108.54	117.95	99.48	3.793	0.427	0.495	0.300

Abbreviations: NH_3_-N, ammonia nitrogen; A/P, Acetate/Propionate ratio; TVFA, total volatile fatty acids. ^a,b^, Means within a row with different superscripts differ significantly (*p* < 0.05).

**Table 4:** Effects of PHDP substitution for oat hay on hematological parameters in Holstein dairy bulls.

Items	Treatment				SEM	*p*-Value		
	T0	T1	T2	T3		Mean	Linear	Quadratic
WBC, 10^9^/L	6.16	8.38	7.27	8.88	0.324	0.054	0.034	0.644
Neu, 10^9^/L	2.16	3.45	2.37	2.91	0.172	0.084	0.469	0.291
Lym, 10^9^/L	3.27	3.88	4.08	4.71	0.183	0.108	0.019	0.975
NLR	0.66	0.9	0.6	0.63	0.048	0.169	0.420	0.31
RBC, 10^12^/L	4.96	5.12	5.5	5.33	0.087	0.184	0.089	0.357
HGB, g/L	87.25	89	91.6	91	1.575	0.756	0.355	0.716
HCT, %	24.5	24.55	25.72	25.23	0.5	0.784	0.479	0.79
MCV, fL	49.53	47.88	46.9	47.3	0.667	0.543	0.233	0.458
MCH, pg	17.68	17.33	16.7	17.05	0.243	0.538	0.282	0.486
PLT, 10^9^/L	211.75	204.5	285.2	369	26.367	0.163	0.04	0.406

Abbreviations: WBC, white blood cell count; Neu, neutrophil count; Lym, lymphocyte count; NLR, neutrophil-to-lymphocyte ratio; RBC, red blood cell count; HGB, hemoglobin concentration; HCT, hematocrit; MCV, mean corpuscular volume; MCH, mean corpuscular hemoglobin; PLT, platelet count.

**Table 5:** Effects of PHDP substitution for oat hay on serum liver and kidney function indices in Holstein dairy bulls.

Items	T0	T1	T2	T3	SEM	*p*-Value
Day30						
TP, g/L	64.08	64.40	77.02	78.47	10.61	0.133
ALB, g/L	28.39	19.69	31.11	29.74	7.75	0.195
GLB, g/L	35.81	44.50	45.80	48.93	11.07	0.352
A/G	0.82	0.45	0.84	0.63	0.43	0.686
GLU	3.83	3.68	2.88	3.26	0.59	0.189
TCHO	0.24	0.34	0.31	0.26	0.11	0.534
TG	2.46	2.23	2.73	1.93	0.76	0.475
CRE, μmol/L	3.83	3.68	2.88	3.26	0.59	0.712
ALT, U/L	20.79 ^a^	12.18 ^b^	19.23 ^a^	20.51 ^a^	3.78	0.020
AST, U/L	56.39 ^a^	38.23 ^b^	53.61 ^a^	62.42 ^a^	8.11	0.010
Day60						
TP, g/L	66.70	88.80	72.46	70.92	14.70	0.446
ALB, g/L	19.86 ^b^	28.68 ^a^	34.96 ^a^	25.01 ^a^	4.81	0.005
GLB, g/L	48.22	52.57	42.38	45.63	12.12	0.767
A/G	0.45	0.51	0.84	0.72	0.28	0.172
GLU	4.68	4.19	3.89	3.87	0.50	0.098
TCHO	0.29	0.31	0.35	0.32	0.10	0.755
TG	2.59	2.22	2.95	2.07	0.58	0.152
CRE, μmol/L	4.68	4.19	3.89	3.87	0.50	0.555
ALT, U/L	24.42 ^a^	18.95 ^b^	25.53 ^a^	21.26 ^a^	3.12	0.032
AST, U/L	57.25	53.90	63.28	60.16	9.08	0.494

Abbreviations: TP, total protein; ALB, albumin; GLB, globulin; A/G, albumin-to-globulin ratio; ALT, alanine aminotransferase; AST, aspartate aminotransferase; GLU, glucose; TCHO, total cholesterol; TG, triglyceride. CRE, creatinine. ^a,b^, Means within a row with different superscripts differ significantly (*p* < 0.05).

**Table 6:** Effects of PHDP substitution for oat hay on serum antioxidant indices in Holstein dairy bulls.

Item	T0	T1	T2	T3	SEM	*p*-Value
Day30						
SOD, U/mL	72.67	55.83	93.67	77.96	23.58	0.177
MDA, nmol/mL	51.06	60.66	55.73	48.04	15.81	0.688
GSH, μmol/L	56.49	54.26	57.78	31.19	15.80	0.095
GSH-PX, U/mL	429.47	489.53	415.53	532.80	184.18	0.833
T-AOC, mmol/L	1.38	1.35	1.37	1.40	0.03	0.156
CAT, U/mL	10.03 ^a^	19.02 ^ab^	33.35 ^b^	40.50 ^b^	9.11	0.001
Day60						
SOD, U/mL	68.55	89.37	96.89	78.41	18.31	0.141
MDA, nmol/mL	40.23	42.88	57.47	46.87	16.12	0.551
GSH, μmol/L	26.09	33.51	27.40	26.39	6.20	0.315
GSH-PX, U/mL	391.24	232.76	288.21	504.98	203.82	0.303
T-AOC, mmol/L	1.39 a	1.36 ^b^	1.40 ^a^	1.41 ^a^	0.01	0.001
CAT, U/mL	38.73	34.57	37.77	41.38	10.43	0.907

Abbreviations: SOD, superoxide dismutase; MDA, malondialdehyde; GSH, glutathione; GSH-PX, glutathione peroxidase; T-AOC, total antioxidant capacity; CAT, catalase. ^a,b^, Means within a row with different superscripts differ significantly (*p* < 0.05).

**Table 7:** Effects of PHDP substitution for oat hay on serum hormone concentrations in Holstein dairy bulls.

Items	T0	T1	T2	T3	SEM	*p*-Value
Day30						
GH, ng/mL	44.51 ^a^	40.08 ^b^	44.85 ^a^	46.44 ^a^	3.26	0.048
IGF-1, ng/mL	1081.32	1076.86	1185.01	1143.58	87.86	0.424
Day60						
GH, ng/mL	36.69	37.63	41.54	39.86	3.85	0.323
IGF-1, ng/mL	1018.99 ^b^	1053.44 ^a^	1051.45 ^a^	1120.52 ^a^	38.52	0.020

Abbreviations: GH, growth hormone; IGF-1, insulin-like growth factor-1. ^a,b^, Means within a row with different superscripts differ significantly (*p* < 0.05).

**Table 8:** Effects of PHDP substitution for oat hay on apparent nutrient digestibility in Holstein dairy bulls (%).

Items	T0	T1	T2	T3	SEM	*p*-Value
Day30						
DM	88.12	88.76	89.51	90.16	2.31	0.593
NDF	89.15	85.26	88.65	87.45	3.49	0.417
ADF	84.76	79.02	83.00	78.78	5.18	0.276
CP	79.92	71.96	78.39	80.05	7.29	0.367
EE	68.78	64.35	58.65	78.66	16.41	0.310
Day60						
DM	90.12	89.59	89.92	90.63	2.65	0.952
ADF	89.38	89.72	89.91	89.83	2.18	0.982
NDF	85.13	85.22	85.21	84.11	3.26	0.950
CP	79.70	79.95	82.43	81.19	4.83	0.807
EE	87.89	89.05	88.70	90.98	2.64	0.395

Abbreviations: DM, dry matter; CP, crude protein; NDF, neutral detergent fiber; ADF, acid detergent fiber; EE, ether extract.

## Data Availability

The datasets generated and analyzed during the current study are available from the corresponding author upon reasonable request.
